# Plasma Microvesicles May Contribute to Muscle Damage in the *mdx* Mouse Model of Duchenne Muscular Dystrophy

**DOI:** 10.3390/ijms26083499

**Published:** 2025-04-08

**Authors:** Cynthia Machado Cascabulho, Samuel Iwao Maia Horita, Daniela Gois Beghini, Rubem Figueiredo Sadok Menna-Barreto, Ana Carolina Heber Max Guimarães Monsores, Alvaro Luiz Bertho, Andrea Henriques-Pons

**Affiliations:** 1 Laboratório de Inovações em Terapias, Ensino e Bioprodutos, Instituto Oswaldo Cruz, FIOCRUZ, Rio de Janeiro 21040-360, RJ, Brazil; samuelhorita@gmail.com (S.I.M.H.); beghini@ioc.fiocruz.br (D.G.B.); anacarol_heber@hotmail.com (A.C.H.M.G.M.); andreah@ioc.fiocruz.br (A.H.-P.); 2 Laboratório de Pesquisas Sobre o Timo, Instituto Oswaldo Cruz, FIOCRUZ, Rio de Janeiro 21040-360, RJ, Brazil; 3 Laboratório de Biologia Celular, Instituto Oswaldo Cruz, FIOCRUZ, Rio de Janeiro 21040-360, RJ, Brazil; rubemb@ioc.fiocruz.br; 4 Laboratório de Imunoparasitologia, Instituto Oswaldo Cruz, FIOCRUZ, Rio de Janeiro 21040-360, RJ, Brazil; alvaro.bertho@ioc.fiocruz.br

**Keywords:** Duchenne muscular dystrophy, *mdx*, microvesicles, extracellular vesicles

## Abstract

Extracellular vesicles (EVs) are cell-derived lipid-bound vesicles divided into apoptotic bodies, microvesicles (MVs), and exosomes based on their biogenesis, release pathway, size, content, and functions. EVs are intercellular mediators that significantly affect muscle diseases such as Duchenne muscular dystrophy (DMD). DMD is a fatal X-linked disorder caused by mutations in the dystrophin gene, leading to muscle degeneration. *Mdx* mice are the most commonly used model to study the disease, and in this study, we phenotypically characterized plasma MVs from *mdx* mice by flow cytometry. Furthermore, we assessed the ability of plasma MVs to modulate muscle inflammation, damage, and/or regeneration by intramuscular injection of MVs from *mdx* mice into *mdx* or DBA/2 mice as a control. In both mouse lineages, platelets and erythrocytes were the primary sources of MVs, and CD3^+^ CD4^+^ MVs were observed only in *mdx* mice. We also observed that plasma MVs from *mdx* mice induced muscle damage in *mdx* mice but not in DBA/2 mice, while plasma MVs from DBA/2 mice did not induce muscle damage in either mouse lineage. These results indicate that plasma MVs from *mdx* are potentially pathogenic. However, this condition also depends on the muscular tissue status, which must be responsive due to active inflammatory or regenerative responses.

## 1. Introduction

Intercellular communication is an essential feature of multicellular organisms and can be mediated through direct cell–cell contact or the transfer of secreted molecules. In recent decades, a third mechanism for intercellular communication involving the intercellular transfer of extracellular vesicles (EVs) has emerged [1]. EVs are spherical lipid-bound structures secreted by cells into the extracellular environment. The three main subtypes of EVs are microvesicles (MVs), exosomes, and apoptotic bodies, which are differentiated based on their biogenesis, release pathways, size, content, and function [2]. Although their formation, size, and biological function may differ, one common point between all vesicles is that they bud from a membrane, whether at the cell surface or from an intracellular vesicular compartment. EVs are known to have a distinctive signature of proteins, lipids, and nucleic acids analogous to their cell or tissue of origin. MVs, also called shedding vesicles, bud directly from the plasma membrane, are 0.1 to 1.0 μm in size, are rich in phosphatidylserine, and contain cytoplasmic cargo [3]. On the other hand, exosomes are multivesicular bodies that arise from the endosomal network [1]. Multiple types of cargo, including proteins, mRNAs, miRNAs, long noncoding RNAs (lncRNAs), and small nucleolar RNAs (SnoRNAs), can interact with MVs and exosomes [1,4,5]. In recent years, EVs have also been shown to affect the course of different muscular diseases, such as muscular atrophy, dematomyositis/polymyositis, and Duchenne muscular dystrophy [6,7,8,9].

Duchenne muscular dystrophy is an X-linked genetic disorder caused by the absence of dystrophin expression, a gene that encodes a 427-kDa protein that connects the cytoskeleton to the sarcolemma and the extracellular matrix. A lack of dystrophin destabilizes muscle fibers during contraction cycles, leading to progressive cell damage through membrane leakage. DMD patients experience symptoms in early childhood and gradually lose mobility during adolescence due to progressive muscle cell atrophy, which is replaced by fibrotic tissue. Patients typically die from cardiorespiratory failure in their third decade of life [10]. Multiple animal models can be used to study DMD, although most frequently, *mdx* mice are used, in which a naturally occurring mutation in the gene encoding dystrophin leads to skeletal muscle weakness, inflammatory infiltration, damage, fibrosis, and late-stage cardiac dysfunction [10].

Plasma EVs are potential biomarkers of DMD, as a set of muscle-enriched miRNAs, named myomiRs (miR-1, miR-133, and miR-206), are highly elevated in the serum of patients with DMD and in dystrophin-deficient animal models. These miRNAs are mainly found in extracellular vesicles and exhibit close to 100% specificity and sensitivity in distinguishing between DMD patients and healthy individuals [11,12]. However, the relationships between muscle pathology and EVs and between muscle pathology and myomiR release are not completely understood. Previous studies have attempted to clarify the role of EVs in the pathogenesis of DMD, and depending on the context, EVs can improve or worsen the disease. Recent work showed that the exposure of control fibroblasts to DMD-derived fibroblast exosomes induced a myofibroblastic phenotype with increased collagen, fibronectin, and α-smooth actin [13]. Using a cardiotoxin-induced necrosis model, it was also shown that the injection of DMD-derived fibroblast exosomes into the mouse tibialis anterior muscle induced greater fibrosis than the injection of control exosomes [13]. Another study reported that injecting EVs secreted by human cardiosphere-derived cells, a heterogeneous population of cardiac cells, into the heart of *mdx* mice augmented cardiac function, ambulatory capacity, and survival [14]. Furthermore, cardiosphere-derived cells and their exosomes transiently and partially restore full-length dystrophin expression in *mdx* mice [14]. Leng et al. demonstrated that repeated systemic administration of exosomes from different sources, mainly murine myotubes, elicited functional rescue and mitigated muscle pathological progression in *mdx* mice without detectable toxicity [15]. Matsuzaka et al. reported that the exposure of C_2_C_12_ myoblasts and myotubes to EVs from the serum of *mdx* mice and the overexpression of miR-133a in C_2_C_12_ cells in the presence of cellular stress resulted in a significant decrease in cell death [16]. The same study also showed reduced muscle degeneration in *mdx* mice treated with an exosome secretion inhibitor, suggesting that extracellular vesicles play a role in worsening the disease [16].

Most studies of EVs in the context of DMD focus on the role of exosomes, and there are few studies on the role of MVs in this disease. In addition, no studies phenotypically characterizing the plasma EVs of *mdx* mice have yet been performed. Therefore, in this work, we aimed to characterize *mdx* plasma MVs and evaluate their impact on muscle damage and regeneration. We observed that, although macrophages are the main cell type composing muscle inflammatory foci in *mdx* mice and DMD patients, macrophages from *mdx* mice released no more MVs than did macrophages from DBA/2 mice. Among T lymphocytes, only CD4^+^ T cells from *mdx* mice released MVs, in addition to platelets and erythrocytes, which are more conventional sources of MVs. After intramuscular adoptive transfer of plasma MVs, we observed that only the autologous combination of plasma MVs from *mdx* mice injected into *mdx* mice induced a muscle response. Therefore, our results indicate that only mice with ongoing muscle inflammatory responses and cycles of degeneration/regeneration are prone to release pathogenic MVs. Moreover, muscle tissue must also be responsive to these biological intercellular mediators. Taken together, we observed that *mdx* plasma MVs are pathogenic, inducing more pronounced cellular inflammatory infiltration and muscle fiber damage.

## 2. Results

### 2.1. MVs from the Plasma of mdx Mice: Quantification, Size, and Morphology Analysis

**Figure 1:**
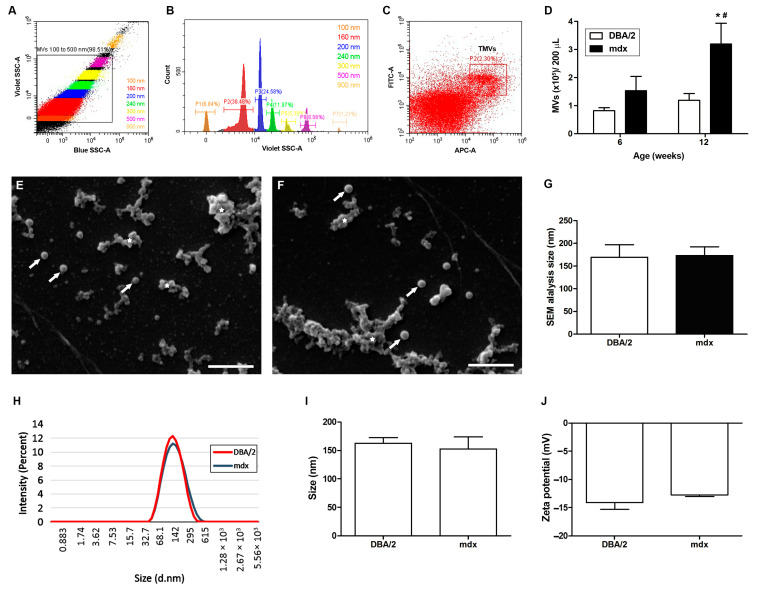
Representative flow cytometry protocol for the detection and quantification of plasma MVs and size evaluation by SEM. The following gating strategy was used: (**A**) dot plot of Blue SSC-log-A vs. Violet SSC-log-A, where a gate encompassing 100–500 nm size MVs was created; (**B**) histogram of Violet SSC-logA, which defines the relative sizes of Megamix FSC and SSC beads. (**C**) Dot plot of calcein AM-FITC-logA vs. annexin APC-log-A, gated on MVs 100–500 nm, where a gate encompassing double-positive events for annexin V/calcein AM was created to determine true MVs (TMV gate). A flow cytometric analytic template was created using CytExpert software v. 2.3 (Beckman Coulter, Brea, CA, USA). (**D**) Bar graphics representing the quantification of MVs from 6- and 12-week-old *mdx* and DBA/2 mice analyzed by flow cytometry. SEM of MVs showing homogeneous vesicle-shaped structures in 12-week-old *mdx* (**E**) and DBA/2 (**F**) mice with sizes ranging from 130 to 210 nm (Bars = 1 µm) (**G**). The white arrows point to MVs, and the white stars indicate clumps of MVs. (**H**) *Mdx* and DBA/2 plasma MVs size-representative curves obtained by DLS. (**I**) Bar graphics representing the size of plasma MVs from 12-week-old *mdx* and DBA/2 mice, determined by DLS. (**J**) Bar graphics representing the Zeta potential of plasma MVs from 12-week-old *mdx* and DBA/2 mice, determined by ELS. * indicates *p* < 0.05 between 12-week-old *mdx* mice and 6-week-old *mdx* mice, and # indicates *p* < 0.05 between 12-week-old *mdx* mice and 12-week-old DBA/2 mice. The data are shown as the means ± SDs; *n* ≥ 4 mice per experiment.

Since there is limited data in the literature regarding plasma MVs from *mdx* mice, we first decided to purify and quantify these structures from *mdx* (D2.B10-Dmd*mdx*/J) and DBA/2 control mice at 6 and 12 weeks of age. At these stages, the muscular inflammatory process is more prominent. This quantification was performed using flow cytometry according to a Blue SSC-log-A vs. Violet SSC-log-A gate encompassing MVs in the range of 100–500 nm (Figure 1A) determined by the relative sizes of the Megamix beads (Figure 1B). Then, the same gate (Figure 1A) was used to evaluate annexin V-APC and calcein AM-FITC double-positive events, called true MVs (TMVs) (Figure 1C). There was no difference in the amount of plasma MVs between DBA/2 and *mdx* mice at 6 weeks of age; however, significantly greater levels of MVs were observed in the plasma of 12-week-old *mdx* mice than in that of DBA/2 mice (Figure 1D).

We next examined the morphology of MVs from 12-week-old *mdx* and DBA/2 mice by scanning electron microscopy (SEM), which revealed that the size of MVs ranged from 130–200 nm in both mouse lineages (Figure 1D,E), with an average of approximately 170 nm (Figure 1F). MV aggregates and clumps were observed via SEM analysis due to the isolation process and the small volume of vesicles (50 µL) needed to perform the assay.

To further characterize *mdx* and DBA/2 plasma MVs and correlate to SEM data, the dynamic light scattering (DLS) technique was employed to measure the diameter of the isolated MVs, and no significant differences were observed in the size of plasma MVs between 12-week-old *mdx* and DBA/2 mice (*mdx* 152.7 nm + 21.02; DBA/2 162.4 nm + 10.21) (Figure 1H,I).

Zeta potential measures the surface charge of EVs and can influence their stability, aggregation state, and interactions with other particles or cells. We evaluated the zeta potential of plasma MVs using electrophoretic light scattering (ELS), and no significant differences were observed between 12-week-old *mdx* and DBA/2 mice (Figure 1J).

### 2.2. Phenotypic Characterization of Plasma MVs from mdx Mice

**Figure 2:**
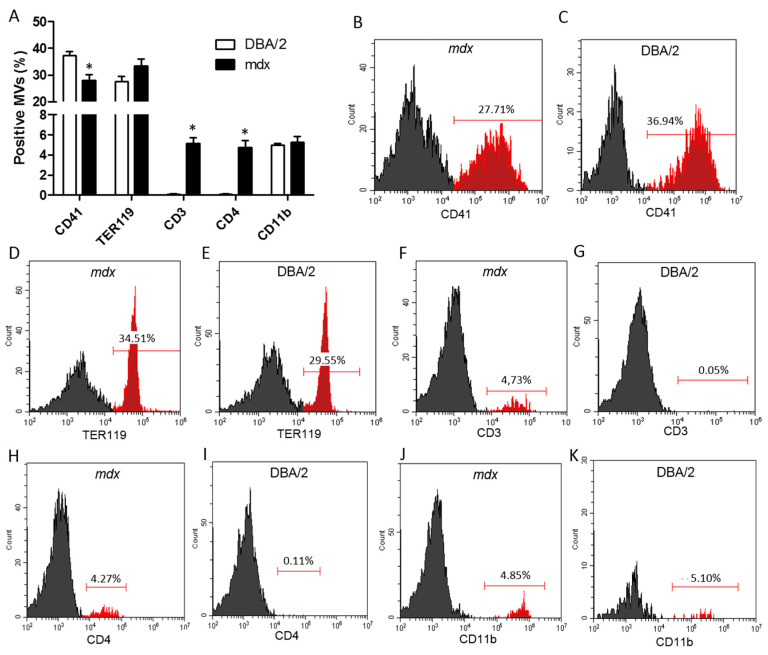
Phenotypic characterization of plasma MVs. Plasma MVs from 12-week-old *mdx* and DBA/2 mice were evaluated for the expression of the following markers inside TMVs: anti--CD41, anti-TER119, anti-CD3, anti-CD4, and anti-CD11b (**A**). The data are shown as the means ± SDs; *n* ≥ 4 mice per experiment. Representative histograms of *mdx* and DBA/2 mouse plasma MVs labeling with anti-CD41 (**B**,**C**), anti-TER119 (**D**,**E**), anti-CD3 (**F**,**G**), anti-CD4 (**H**,**I**), and anti-CD11b (**J**,**K**) are shown in the figure. * indicates *p* < 0.05 between *mdx* and DBA/2 mice. One-way ANOVA followed by Tukey’s post hoc test was used.

Since MVs have a signature repertoire of proteins analogous to their cell or tissue of origin, we evaluated their phenotype in the plasma of *mdx* and DBA/2 mice. Because the majority of plasma MVs are derived from platelets and erythrocytes, we analyzed the expression of CD41a (platelet-derived) and TER-119 (erythrocyte-derived) in plasma MVs from 12-week-old *mdx* and DBA/2 mice (Figure 2A–E). Although a large percentage of MVs in both mouse lineages express either molecule, the frequency of CD41^+^ MVs and the median of fluorescence intensity (MFI) were lower in *mdx* mice than in DBA/2 mice (Figure 2A–E). Then, we evaluated MVs from T lymphocytes (CD3^+^, CD4^+^, or CD8^+^), B lymphocytes (CD19^+^) and general myeloid cells (CD11b^+^). There was a 47-fold increase in the percentage of CD3^+^ cells (Figure 2A,F,G) and a 38.8-fold increase in the percentage of CD4^+^ MVs (Figure 2A,H,I) in *mdx* mice compared to DBA/2 mice. There was no significant difference in the percentage of TER119 or CD11b^+^ cells (Figure 2A,D,E,J,K) between *mdx* mice and DBA/2 mice. 

MiRNAs are known to play a role in intercellular communication mediated by EVs, as they can be internalized by recipient cells and influence their gene expression. Therefore, we evaluated whether there is a modulation in the concentration of nucleic acids in the plasma MVs of *mdx* mice. We then incubated plasma MVs with thiazole orange, a compound that binds to both RNA and DNA and forms a green fluorescent nucleotide/reagent complex. We observed that the MFI of plasma MVs from *mdx* mice was 3.2-fold greater than that of plasma MVs from DBA/2 control mice, indicating that the amount of nucleic acid cargo in MVs from *mdx* mice was significantly greater (Supplementary Figure S1).

### 2.3. Quantification of Plasma MVs from mdx Mice after Spontaneous Physical Activity

**Figure 3:**
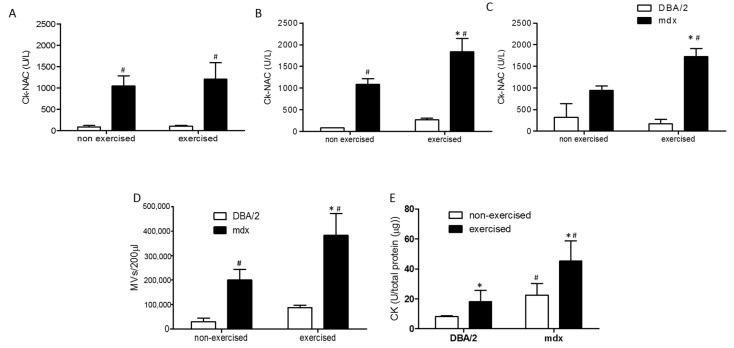
Plasma MV evaluation after the swimming protocol. CK-NAC activity was evaluated in the plasma of *mdx* and DBA/2 mice at the end of the first (**A**), second (**B**), and third (**C**) weeks of the swimming protocol. (**D**) Plasma MV quantification in exercised or nonexercised *mdx* and DBA/2 mice. The data are shown as the means ± SDs; *n* ≥ 5 mice; * indicates *p* < 0.05 between exercised and nonexercised mice of the same lineage; # indicates *p* < 0.05 between *mdx* and DBA/2 mice subjected to exercise and between *mdx* and DBA/2 nonexercised mice. One-way ANOVA followed by Tukey’s post hoc test was used. (**E**) CK-NAC activity in plasma MVs from *mdx* and DBA/2 mice subjected to exercise or not subjected to exercise. The data are shown as medians with ranges; three experiments were carried out with a sample pool of 10 mice in each experiment; * indicates *p* < 0.05 between exercised and nonexercised mice of the same lineage, and # indicates *p* < 0.05 between *mdx* and DBA/2 mice subjected to exercise and between *mdx* and DBA/2 nonexercised mice. The Kruskal–Wallis test, followed by Dunn’s post hoc test, was used.

Since *mdx* mice have a milder DMD phenotype than humans, treadmill or swimming exercises can be employed to induce muscle damage and worsen the *mdx* phenotype. We quantified MVs in the plasma of 12-week-old *mdx* and DBA/2 mice after induced swimming. The exercised groups were subjected to 20 min of swimming five days per week for three weeks. At the end of each week, blood was collected from the tail snips to evaluate CK-NAC as an indicator of muscle damage. At the end of the first week of exercise, no difference in CK-NAC activity was observed between exercised and nonexercised *mdx* mice (Figure 3A) or between exercised and nonexercised DBA/2 mice (Figure 3A). As expected and described in the literature, compared with DBA/2 mice, exercised and nonexercised *mdx* mice showed a significant increase in CK-NAC (Figure 3A). At the end of the second (Figure 3B) and third (Figure 3C) weeks of exercise, a significant increase in CK-NAC was observed in the exercised *mdx* mice compared to the nonexercised mice. Moreover, no difference was observed between exercised and nonexercised DBA/2 mice (Figure 3B,C).

Since there was an exacerbation of muscle damage after exercise, we evaluated whether there was an increase in the concentration of plasma MVs after swimming. At the end of the third week, blood was collected by cardiac puncture, and the plasma MV concentration was 3.5-fold greater in exercised *mdx* mice than in nonexercised *mdx* mice (Figure 3D). Although we did not observe an increase in CK-NAC after three weeks of swimming, indicating no muscle damage after exercise, a 2-fold increase in the concentration of plasma MVs was observed after swimming (Figure 3D). Therefore, we tested whether these MVs were of muscular origin. To answer this question, we measured CK-NAC in the plasma MVs of exercised and nonexercised *mdx* and DBA/2 mice, and the results were normalized to the amount of total protein in the sample (Figure 3E). A 2-fold increase in CK-NAC concentration was detected in the plasma MVs of exercised *mdx* mice compared with those of nonexercised mice, and a 2.3-fold increase was detected in the plasma MVs of exercised DBA/2 mice compared with those of nonexercised DBA/2 mice (Figure 3E). Plasma MVs from nonexercised *mdx* mice had 3.2× more CK-NAC than those from nonexercised DBA/2 mice, and plasma MVs from exercised *mdx* mice had 2.6× more CK-NAC than those from exercised DBA/2 mice (Figure 3E).

### 2.4. Analysis of the Fiber Size Distribution and Inflammatory Response after In Vivo Transfer of Plasma MVs

**Figure 4:**
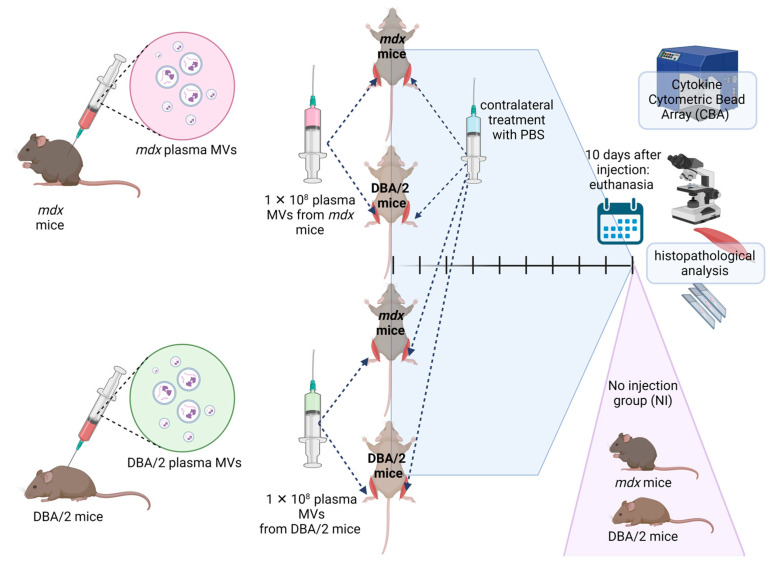
Schematic representation of the plasma MV transfer protocol. Plasma MVs were obtained from 12-week-old *mdx* and DBA/2 mice, and a total of 1 × 10^8^ MVs were injected into the right TA muscle paw of each animal. The following groups were formed: *Mdx* mice that received MVs from *mdx* mice, *mdx* mice that received MVs from DBA/2 mice, DBA/2 mice that received MVs from DBA/2 mice, and DBA/2 mice that received MVs from *mdx* mice. The left tibialis anterior paw of mice that received only PBS and a group of mice (*mdx* and DBA/2) that received no injection were also included (NI). Ten days after the injection, the animals were euthanized, and the tibialis anterior muscles were collected for histopathological analysis and cytokine measurement via flow cytometry. Figure created with BioRender.com.

A plasma MV transfer assay was performed to evaluate the role of plasma MVs in muscle damage or regeneration *in vivo*. In this assay, MVs were obtained from 12-week-old *mdx* and DBA/2 mice not subjected to exercise and injected into the right paw tibialis anterior (TA) of 12-week-old *mdx* and DBA/2 recipient mice. Therefore, the four groups were as follows: *mdx* mice that received MVs from *mdx* mice (*mdx* MVs *mdx*), *mdx* mice that received MVs from DBA/2 mice (*mdx* MVs DBA/2), DBA/2 mice that received MVs from DBA/2 mice (DBA/2 MVs DBA/2), and DBA/2 mice that received MVs from *mdx* mice (DBA/2 MVs *mdx*). The contralateral left paw of each animal received only PBS (*mdx* PBS or DBA/2 PBS), and a fifth group of animals received no injections (NI). Ten days post-injection, the TA muscles were collected and processed, and histopathological analysis and cytokine measurements were conducted (Figure 4).

**Figure 5:**
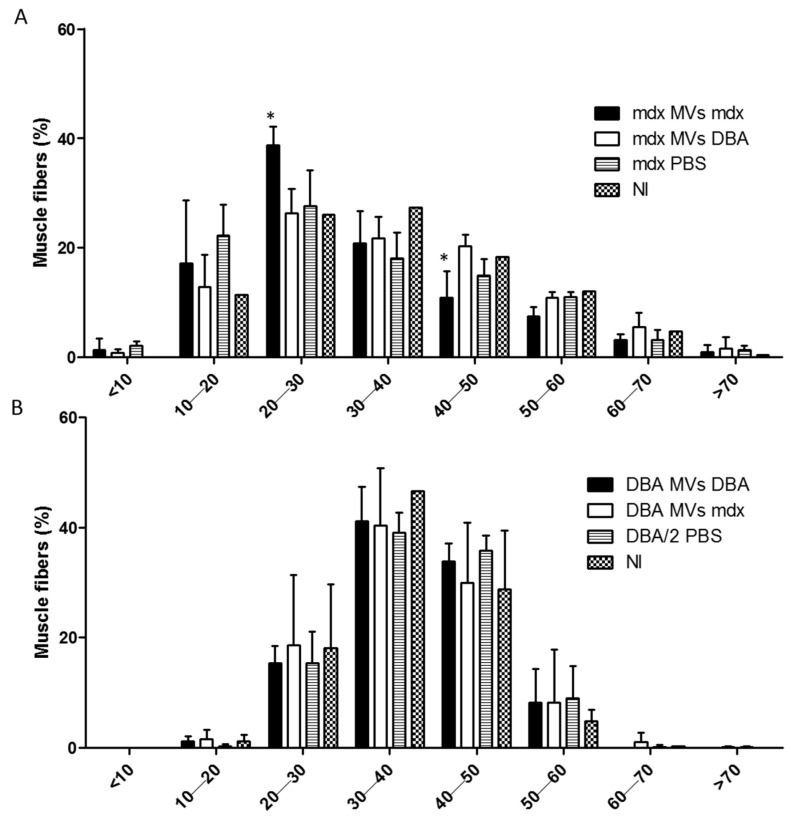
Analysis of the minimal Feret diameter in *mdx* and DBA/2 mice after plasma MV injection. (**A**) Mean minimal Feret diameter determined in the TA of *mdx* mice that received plasma MVs from *mdx* mice, DBA/2 mice, only PBS, or no injection (NI). (**B**) The mean minimal Feret diameter was determined in the TA of DBA/2 mice that received plasma MVs from DBA/2 mice, *mdx* mice, mice receiving only PBS, or mice that received no injection. The data are shown as the means ± SDs; *n* ≥ 4 mice; * indicates *p* < 0.05 for comparisons between *mdx* mice that received plasma MVs from *mdx* mice and the other *mdx* groups. One-way ANOVA followed by Tukey’s post hoc test was used.

First, we determined the minimal Feret diameter, which is a morphometric parameter used in order to have the least bias when measuring muscle fiber diameter due to the angle orientation of the tissue section and is considered the most robust parameter when measuring muscle fiber size. This analysis, plotted according to the fiber size distribution, revealed a shift toward smaller myofibers in the TA muscle in the *mdx* MV *mdx* group than in the *mdx* MV DBA, *mdx* PBS, and NI groups (Figure 5A). *Mdx* mice that received *mdx* plasma MVs had significantly more myofibers less than 20 to 30 μm in diameter on average than did the other groups (*mdx* MVs *mdx*: 38.77 ± 3.4%; *mdx* MVs DBA/2: 25.33 ± 4.3%; *mdx* PBS: 27.55 ± 6.5%; NI: 24.06 ± 2.8%, *p* ≤ 0.05) (Figure 5A). Moreover, the number of larger myofibers between 40 and 50 μm in diameter was significantly lower in the *mdx* mice that received MVs from *mdx* mice than in the other groups (*mdx* MVs *mdx* 10.88 ± 4.8% fibers in the range of 40 to 50 μm; *mdx* MVs DBA/2 20.33 ± 3.08%; *mdx* PBS 16.88 ± 2.98%; and NI 18.33 + 2.58) (Figure 5A). When the DBA/2 mice received MVs, even from *mdx* mice, no significant changes were observed in the fiber size distribution among any of the groups evaluated (Figure 5B).

**Figure 6:**
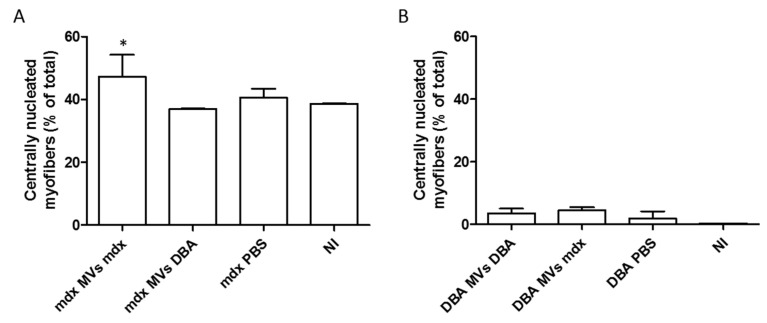
Analysis of centrally nucleated myofibers in *mdx* and DBA/2 mice after plasma MV injection. The percentage of muscle fibers with centrally nucleated myofibers was determined in the TAs of *mdx* mice that received plasma MVs from *mdx* mice, DBA/2 mice, only PBS, or no injection (NI) (**A**). The percentage of muscle fibers with centrally nucleated myofibers was determined in the TAs of DBA/2 mice that received plasma MVs from DBA/2 mice, *mdx* mice, only PBS, or no injection (NI) (**B**). The data are shown as medians with ranges; * indicates *p* < 0.05 between *mdx* mice that received plasma MVs from *mdx* mice and the other *mdx* groups. The Kruskal–Wallis test, followed by Dunn’s post hoc test, was used.

Another parameter analyzed was the percentage of fibers with central nucleation since this event is related to muscle damage and is commonly observed during muscle regeneration. The results revealed a significantly greater percentage of centrally nucleated myofibers in the *mdx* MVs *mdx* group (49.21% + 4.37) than in the *mdx* MVs DBA (36.34% + 1.19), *mdx* PBS (38.96 + 5.4), and NI (37.91% + 1.35) groups (Figure 6A). On the other hand, centrally nucleated myofibers were rarely observed in DBA/2 mice, where they represented at most 5% of the total muscle fibers analyzed (Figure 6B).

**Figure 7:**
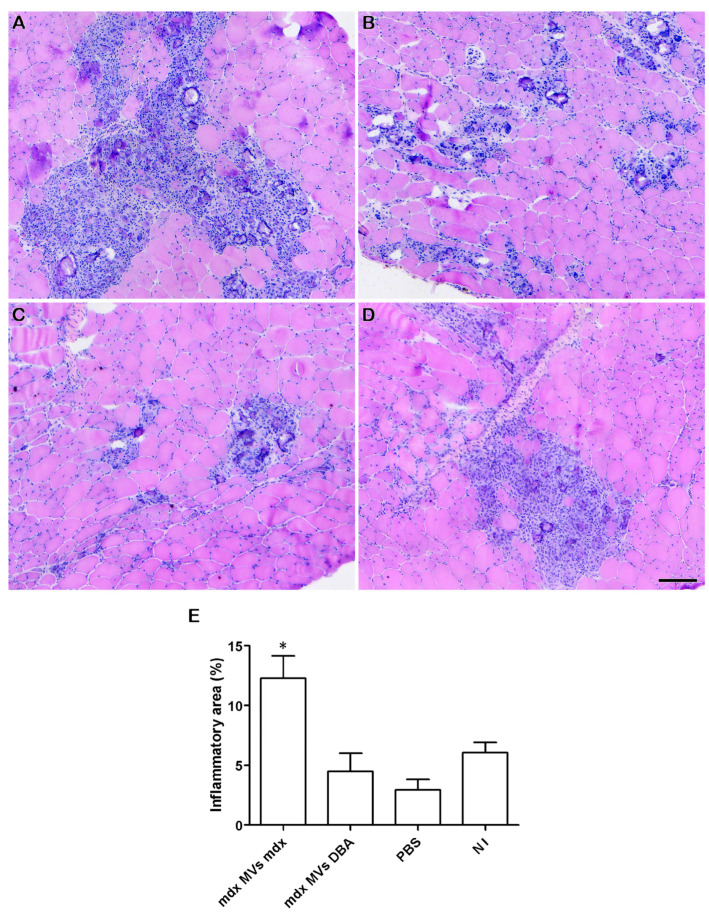
Analysis of the inflammatory area in the TA of *mdx* mice after injection of plasma MVs. Representative images of inflammatory infiltrates in the TAs of *mdx* mice that received plasma MVs from *mdx* mice (**A**), DBA/2 mice (**B**), mice that received only PBS (**C**), and mice that received no injection (**D**). (**E**) Percentage of tissue area occupied by inflammatory infiltrates in *mdx* mice that received plasma MVs from *mdx* mice, DBA/2 mice, only PBS, or no injection. Bars = 120 µm, and * indicates *p* < 0.05. One-way ANOVA followed by Tukey’s post hoc test was used.

**Figure 8:**
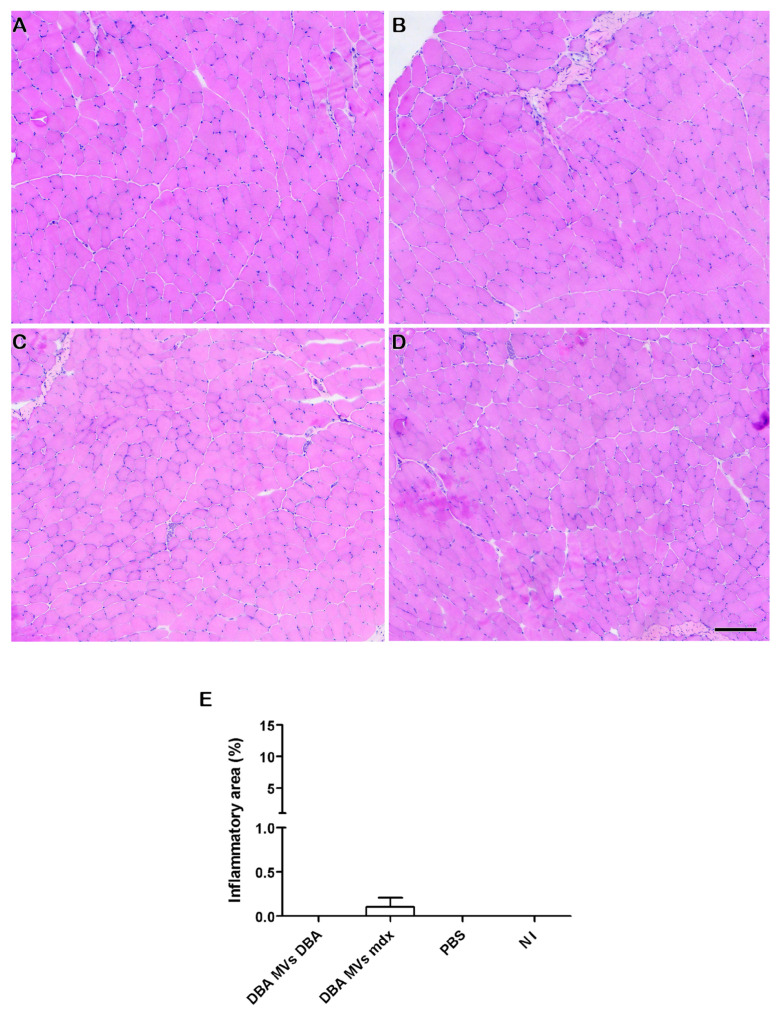
Analysis of the inflammatory area in the TA of DBA/2 mice after injection of plasma MVs. Representative images of inflammatory infiltrates in the TAs of DBA/2 mice that received plasma MVs from DBA/2 mice (**A**), *mdx* mice (**B**), mice that received only PBS (**C**), and mice that received no injection (**D**). (**E**) Percentage of tissue area occupied by inflammatory infiltrates in DBA/2 mice that received plasma MVs from DBA/2 mice, *mdx* mice, only PBS, or no injection. Bars = 120 µm.

Since inflammation is an important hallmark in DMD skeletal muscles, inflammatory infiltrates in the TA muscle after injection of MVs were evaluated. Greater inflammatory infiltrates were observed in the *mdx* MVs *mdx* group (Figure 7A) than in the *mdx* MVs DBA (Figure 7B), *mdx* PBS (Figure 7C), and NI (Figure 7D) groups. The percentage of tissue occupied by inflammatory foci was significantly greater in the *mdx* MVs *mdx* group (12.29% + 3.73) than in the *mdx* MVs DBA (4.48% + 2.61), *mdx* PBS (2.94 + 1.49), and NI (6.04 + 1.24) groups. When DBA/2 mice received MVs, no inflammatory foci were observed in any of the groups (Figure 8).

**Figure 9:**
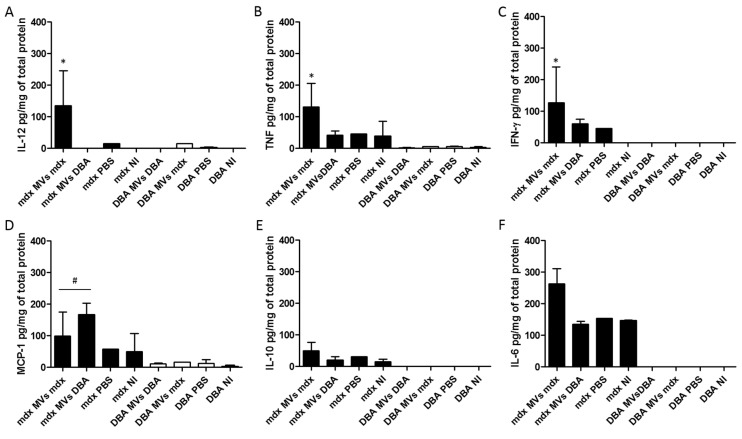
Cytokine profile in the TA of *mdx* and DBA/2 mice after the plasma MV transfer protocol. TA extracts from *mdx* and DBA/2 mice that received plasma MVs from *mdx* mice, DBA/2 mice, only PBS, or no injection were used to measure IL-12 (**A**), TNF (**B**), IL-6 (**C**), MCP-1 (**D**), IFN-γ (**E**), and IL-10 (**F**) by flow cytometry using a cytokine bead array assay. The data were normalized to the concentration of total proteins per sample. The data are shown as medians with ranges; * indicates *p* < 0.05 between *mdx* mice that received plasma MVs from *mdx* mice and the other *mdx* groups, and # indicates *p* < 0.05 between *mdx* mice that received plasma MVs from *mdx* mice and *mdx* mice that received plasma MVs from DBA/2 mice and the other *mdx* groups. The Kruskal–Wallis test, followed by Dunn’s post hoc test, was used.

Since a significant increase in the inflammatory area was observed only in the *mdx* MVs *mdx* group, we evaluated the cytokine profile in the TA after the transfer of MVs. We observed a significant increase in IL-12 (Figure 9A), TNF (Figure 9B), and IL-6 (Figure 9C) in the *mdx* group compared to the other groups. The MCP-1 levels (Figure 9D) in the *mdx* MVs *mdx* and *mdx* MVs DBA groups were significantly greater than those in the *mdx* PBS and *mdx* NI groups. No changes in IFN-γ or IL-10 cytokine levels were observed among the *mdx* mouse groups. In the DBA mouse groups, no alterations in cytokine levels were detected.

## 3. Discussion

Although MVs play important roles in various physiological and pathological processes, such as immune response modulation, tissue repair, cancer progression, cardiovascular disease, thrombosis, and diabetes, MVs have only recently emerged as potential players in the pathogenesis and progression of DMD [12]. EVs secreted by skeletal muscles carry a wide range of myokines, miRNAs, proteins, and mRNAs that are thought to play various roles in muscle homeostasis, development, and myogenesis [17]. Since EV cargo plays a significant role in the musculoskeletal system, in this work, we aimed to quantify and phenotypically characterize plasma MVs from *mdx* mice and evaluate their ability to modulate muscle pathology. To the best of our knowledge, this is the first time that MVs have been quantified in the plasma of *mdx* mice. We observed a significant increase in the concentration of plasma MVs in 12-week-old *mdx* mice compared to DBA/2 control mice of the same age, which is consistent with the fact that the release of EVs is increased under cellular stress [18]. Using three technical approaches (flow cytometry, SEM, and DLS), we verified that in our isolation protocol, we obtained MVs with a size compatible with that described in the literature.

Methods for studying EVs have been extensively developed and advanced in recent years. The tremendous biological impact of EVs has driven many researchers to improve the techniques and methods of extraction, obtain a specific and pure vesicular population from the biological matrix, and achieve reproducibility of isolation [19]. The choice of separation method and subsequent concentration of the isolated vesicular particles depends on factors varying from one study to another. In our research, we opted for an isolation protocol that prioritized microvesicles over exosomes since we used 20,000× *g* centrifugation of platelet-free plasma to obtain them, as described elsewhere [20,21]. Several methods of EV quantification exist, including resistive pulse sensing [22], microscopy (scanning/transmission electron microscopy and atomic force microscopy) [23,24], dynamic light scattering (DLS) [25], nanoparticle tracking analysis (NTA) [26], flow cytometry [27], and small-angle X-ray scattering (SAXS). With advances in flow cytometry, especially in technology improving the nanometric sensitivity of flow cytometers, this methodology has become a fundamental tool for detecting and phenotyping MVs in almost all body fluids (e.g., plasma, urine, and saliva, among others) and has significant advantages over other single-particle detection techniques [28]. Among these advantages, greater accuracy in determining the concentration and diameter of particles, as well as in the phenotypic characterization of EVs labeled with fluorescent molecules, are particularly important for this study [28]. The nanoflow cytometer used in the current study was a CytoFLEX S (Beckman Coulter) instrument equipped with 405 nm, 488 nm, and 638 nm lasers to detect up to twelve fluorescence, FSC, and three SSC parameters. In conventional flow cytometry, MVs and other biological nanoparticles are detected within the debris region, and most flow cytometers are not sensitive enough to effectively detect particles smaller than 300 nm in diameter [29]. Flow cytometers sensitive to this purpose include APD sensors and a Violet SSC detector, which has an attenuation filter to reduce 95% of the scatter signal and adequately increase the sensitivity to the nanoparticle range [30]. A shorter wavelength, such as 405 nm (violet), results in more significant orthogonal particle light scattering and increases the resolution range. The refractive indices of nanometric particles such as MVs and viruses are amplified using a violet laser, thereby improving their detection [30].

Since phosphatidylserine (PS) is translocated to the outer membrane during microvesicle biogenesis, many studies have used annexin V as a marker of MVs. Nevertheless, it is known that annexin V can bind to PS found on disrupted MVs and/or MV debris, leading to a misinterpretation of false-positive events. Labeling with calcein-AM can aid in avoiding this problem. Calcein AM is a membrane-permeable labeling dye with an acetoxymethyl ester (AM) group that is cleaved by esterase upon entering the MV, yielding the membrane-impermeable calcein fluorescent dye. MVs with compromised cell membranes do not retain calcein, preventing misinterpretation between intact/true MVs and debris labeling. Therefore, true MVs are those that are double positive for annexin V and calcein [19].

Platelets are the primary source of EVs in the blood. They are produced upon activation and are associated with noninfectious chronic [31] and infectious parasitic inflammatory diseases [29]. In fact, in our flow cytometry experiments, we observed a high percentage of CD41a^+^ MVs in both 12-week-old *mdx* mice and DBA/2 control mice, although this percentage was significantly lower in *mdx* mice than in DBA/2 mice. Schorling et al. showed that platelets from patients with DMD have decreased expression of CD62P and CD63, which are markers of platelet activation, and this could be related to a reduced release of MVs in mice [32]. Indeed, in our work, we observed a reduction in the percentage of CD41^+^ MVs in *mdx* mice compared to DBA/2 mice. Another study showed that platelet-derived growth factor BB (PDGF-BB), a molecule found in platelet-derived EVs, may play a protective role in muscular dystrophies by enhancing muscle regeneration through satellite cell activation, proliferation, and migration [33]. Our data, in contrast, showed that even with a high percentage of platelet-derived MVs, plasma MVs from *mdx* mice exacerbate the muscle damage observed in this lineage.

Erythrocytes are the most abundant cell type in blood and could represent an essential source of EVs. These vesicles may form during circulating erythrocyte aging due to complement-mediated calcium influx, plasma membrane budding, and subsequent vesicle shedding [34]. Red blood cell-derived EVs (RBCEVs) are critical for communicating with endothelial cells to regulate NO and O_2_ homeostasis and can affect many immune cells [35]. In addition, they can promote the production of proinflammatory cytokines (IL-2, IL-7, IL-15, and TNF) by interacting with macrophages and increasing the proliferation of CD4^+^ and CD8^+^ T cells by influencing antigen-presenting cells [36]. RBCEVs are essential for the pathogenesis and progression of cardiovascular and related metabolic diseases. In addition, RBCEVs enhance tumor necrosis factor (TNF) production in monocytes, increase mitogen-induced CD4^+^ and CD8^+^ T-cell proliferation, and induce endothelial cell apoptosis, leading to vascular dysfunction [37]. However, despite their relevant role in several pathologies, further studies are still needed to evaluate RBCEVs in DMD or animal models of the disease. In our study, no differences in the percentage of erythrocyte-derived MVs were detected between *mdx* and DBA/2 mice.

We observed the presence of CD3^+^ and CD4^+^ plasma MVs in *mdx* mice, but the same effect was not observed in control DBA/2 mice. Although the number of studies on T-cell-derived vesicles is limited, some studies have shown that these vesicles could play an important role in immune regulation. The secretion of EVs by T cells was shown to be triggered by TCR activation, and these EVs could transfer TCR and MHC-II molecules to B cells and dendritic cells, modulating their antigen-presenting cell function [38]. The dysregulated release of lymphocyte-derived EVs has been implicated in various pathological conditions, including autoimmune diseases, infectious diseases, cancer, and inflammatory disorders [39,40,41,42]. These vesicles can exacerbate disease progression by promoting inflammation and immune evasion. Our results show that the nature of the circulating MVs in *mdx* mice is much different from that in control DBA/2 mice, as activated CD4^+^ T lymphocytes may be shedding MVs. It is interesting, however, that CD8^+^ T lymphocytes released no detectable levels of MVs.

Although cellular inflammatory infiltration acts as a secondary inducer of muscle damage and fibrosis in patients and *mdx* model animals, our understanding of immune cell-derived EVs is still limited. Although macrophages are the primary inflammatory cells found in the skeletal muscles of DMD patients and *mdx* mice [43], we did not find F4/80^+^ MVs in either the *mdx* or DBA/2 control mice.

Once released into the extracellular space, EVs can reach recipient cells and deliver their contents, mainly proteins, small RNAs (sRNAs), tRNA fragments, and miRNAs, thereby regulating recipient cell gene expression and function. Matsuzaka et al. detected increased levels of muscle miRNAs, namely, the myomiRs miR-1, miR-133a, and miR-206, in the sera of DMD patients and *mdx* mice [16]. Our work did not quantify or identify the nature of RNA in the plasma MVs of *mdx* mice. However, using the thiazole orange dye, we observed an increase in the MFI in the plasma MVs of *mdx* mice compared to those of DBA/2 mice by flow cytometry. Therefore, the ability of *mdx*-derived MVs to transport nucleic acids is increased.

The release of EVs into circulation is particularly enhanced by physical exercise, inflammation/stress, and several muscle-related conditions [44,45]. Brisamar et al. showed that rat EV levels increased considerably immediately after exercise but remained unchanged after four weeks of swimming-based training [46]. In our study, compared with nonexercised *mdx* mice, exercised *mdx* mice had a 1.9-fold increase in the concentration of plasma MVs and a 38-fold increase in the concentration of plasma MVs after three weeks of swimming-based training. An increase in the concentration of plasma MVs after swimming was also observed in DBA/2 mice, although in a smaller proportion than in exercised *mdx* mice. This result agrees with what is observed in DMD patients who have increased muscle damage after physical activity. This effect has always been associated with mechanical damage induced by membrane leakage after cycles of muscle fiber contraction. However, the MVs released may also contribute to this increased damage after muscle activity.

In our experiments, compared with those of nonexercised *mdx* mice, MVs from exercised mdx mice showed increased CK-NAC enzyme activity, indicating the presence of MVs of muscular origin in their plasma. A recent study using rats suggested that the release of EVs carrying miR-1, a conserved miRNA highly expressed in muscles, increased after swimming. Moreover, the expression of other muscle miRNAs in MVs, such as miR-133a, miR-133b, and miR-206, increased immediately after but returned to the baseline level after 48 h [46]. Notably, plasma MVs from nonexercised *mdx* mice already showed increased CK-NAC activity compared to MVs from nonexercised DBA/2 mice. These data indicate that regardless of physical activity, these animals already have a higher concentration of MVs of muscular origin, probably due to the natural muscle damage generated by the disease.

Several lines of evidence have shown that EVs are key players in muscle physiology and pathophysiological processes. Sahu et al. showed that serum from young mice restored the bioenergetic and myogenic profiles of aged muscle cell progeny and that this effect depended on circulating EVs [47]. In vivo experiments confirmed that young mouse serum enhanced muscle regeneration in young mice and that this effect was diminished when the serum was depleted of EVs [47]. Another study showed that atrophic muscle fiber-derived EV miR-690 inhibited satellite cell differentiation during aging-induced muscle atrophy [48]. Choi et al. demonstrated that treating wounded muscle with muscle-derived EVs reduced fibrosis and increased myofiber regeneration [49]. It was also shown that incubation of myoblasts with EVs released from myotubes pretreated with hydrogen peroxide led to a significant reduction in the diameter of myotubes and stimulation of myoblast proliferation [50]. These data, taken together, show that, depending on the context, EVs can favor or inhibit muscle regeneration.

In our work, we observed that plasma MVs from *mdx* mice induced muscle damage when injected into the TA of *mdx* mice but not when injected into the TA of DBA/2 control mice. Moreover, MVs from DBA mice did not induce muscle damage in *mdx* mice. These results indicate that plasma MVs from *mdx* are potentially pathogenic, and the nature of the MV content and the recipient’s muscle environment are important for the pathogenic response. Due to the lack of dystrophin, *mdx* myofibers are constantly degenerating and regenerating with destabilized plasma membranes. It is possible that degenerating myofibers, which are more permeable to injected dyes, such as Evans blue and endogenous IgM, are prone to receiving a more significant load of MVs. In this case, invading MVs could act as pathogenic inducers of muscle damage and secondary inflammation. Our results are in agreement with those of Matsuzaka et al., who reported reduced muscle degeneration in *mdx* mice treated with an exosome secretion inhibitor, indicating that extracellular vesicles play a role in worsening the disease [16]. However, MV-dependent muscle degeneration may be indirect, with MVs not leading to muscle fiber damage but rather acting on pathogenic inflammatory cells. In this case, MV-stimulated macrophages are more likely to be cytotoxic to muscle fibers, which are the main cell type in the muscles of *mdx* mice. This possibility remains to be tested in vitro.

Regarding the muscular inflammatory environment after MV transfer, we observed a significant increase in the levels of IL-6, TNF, and IL-12 in the TA of *mdx* mice that received MVs derived from *mdx* mice compared to those in the other groups, consistent with the exacerbation of muscle damage observed via histopathological analyses. Wada et al. reported reduced fibrosis and a significant increase in the area of embryonic myosin heavy chain-positive myofibers and muscle diameter in dystrophin/utrophin double-knockout mice treated with anti-IL-6 [51]. In addition, Pelosi et al. showed that *mdx* mice overexpressing IL-6 present an exacerbated dystrophic muscular phenotype similar to that observed in DMD patients [52]. These findings suggest that IL-6 has a role in worsening this disease.

Plasma samples obtained from resting *mdx* mice showed that CD4^+^ T cells but not CD8^+^ T or B lymphocytes or macrophages contributed significantly to the MVs observed. Although macrophages are the primary muscle inflammatory foci in *mdx* mice and DMD patients, these cells apparently do not release MVs into the extracellular environment. Our results indicate that muscle-, CD4^+^ T lymphocyte-, and possibly platelet-derived MVs positively modulate muscle inflammatory infiltration, which leads to increased muscle damage and regeneration. The constant cycles of muscle fiber degeneration and regeneration due to sarcolemma mechanical leakage and secondary damage induced by inflammatory cells are likely enhanced by MVs.

## 4. Materials and Methods

### 4.1. Animals

All experiments were conducted using mice from the Center for Breeding of Laboratory Animals (ICTB) at Fundação Oswaldo Cruz. We used 6- and 12-week-old male *mdx* mice (D2. B10-Dmd*mdx*/J) and age-matched DBA/2/J control mice. All mice were housed for at least one week before experimentation under conditions complying with the “Guide for the Care and Use of Laboratory Animals” (DHEW Publication No. [NIH] 80–23, 1996). The FIOCRUZ Committee of Ethics in Research approved this project (L020/2019-A1) according to resolution 196/96 of the National Health Council of the Brazilian Ministry of Health.

### 4.2. Swimming Protocol

To induce muscle fiber damage, mice were forced to swim for 20 min five days per week for three weeks. The mice were placed inside a Plexiglass box filled with warm water (28 °C), and any contact with the bottom of the box was avoided.

### 4.3. Blood Collection and MV Isolation through Serial Centrifugation

Currently, there are some standardized flow cytometry protocols for analyzing EV plasma samples, but they still contain some controversial points. Some issues, mainly in preanalytical procedures (e.g., blood collection and delay time between collection and sample processing/analysis), need to be standardized to the start of analysis by flow cytometry. Thus, we adapted and standardized the preanalytical and sample processing procedures based on those described elsewhere and reported according to the criteria established by the MISEV guidelines [28,53,54]. Briefly, 1 mL blood samples were collected by cardiac puncture using a 21G × 1 ¼″ needle to minimize platelet activation and hemolysis in the presence of sodium citrate 0.109 M. Sample tubes were carefully transported vertically at room temperature without agitation. Within 30 min of blood withdrawal, platelet-depleted plasma was prepared as follows: centrifugation at 300× *g* for 10 min at 20 °C, with acceleration and braking to obtain the plasma (supernatant); centrifugation of the supernatant at 2500× *g* for 15 min at 20 °C, with the lowest deceleration, to obtain platelet-poor plasma (PPP). Subsequently, the PPP was gently transferred into a polypropylene tube and centrifuged at 13,000× *g* for 5 min at 20 °C with acceleration and braking to obtain platelet-free plasma (PFP). The PFP was gently transferred into another tube and centrifuged at 20,000× *g* for 20 min at 20 °C. The supernatant was discarded, and the pellet was resuspended in 2 mL of annexin binding buffer (BD Biosciences, Franklin Lakes, NJ, USA) previously filtered through a 0.2 μm mesh to reduce background noise during flow cytometry analysis. The samples were always freshly analyzed and never subjected to any round of freezing/thawing. Samples with detectable hemolysis were discarded. We did not fast the animals before the blood collection. We aimed to reproduce human disease and MV formation as much as possible under normal conditions without nutritional stress.

### 4.4. Plasma MV Quantification and Phenotyping

For MV concentration determination and phenotyping, mouse plasma samples were subjected to a flow cytometric labeling protocol consisting of the following labeling panels: annexin V-APC (BD Biosciences, Franklin Lakes, NJ, USA); calcein AM (Thermo Fisher Scientific, Waltham, MA, USA) supplemented with Panels 1-anti-CD41 PerCP, TER119-AlexaFluor700, anti-CD11b-PE, and anti-CD3-PECy7; or Panels 2-anti-CD19 PECF594, anti-CD4 PE, anti-CD8 APC-Cy7, and anti-F4/80 PECF594 (all from Biolegend, San Diego, CA, USA). Before staining, the antibody mixture was centrifuged at 20,000× *g* for 30 min to remove protein aggregates. All the samples were incubated for 30 min at 37 °C, after which they were diluted with 2 mL of annexin binding buffer and analyzed with a Cytoflex S nanoflow cytometer (Beckman Coulter, Brea, CA, USA) equipped with 405 nm, 488 nm, 561 nm, and 638 nm lasers.

To adjust CytoFlex for MV measurements, the standard filter configuration was changed so that the Violet SSC was used as a trigger signal to discriminate the noise instead of the normally used 488 nm FSC. The changes in the filter configuration used to detect MVs were as follows: in front of the 405 nm laser APD sensors, the 450/45 nm filter position was changed to 405/10 nm, the 525/40 nm filter position was changed to 450/45 nm, and the 610/20 nm filter position was changed to 525/40 nm. All the lasers used for filtering from 488 nm to 638 nm remained in the default-conventional CytoFlex setup. To reduce the background noise, this flow cytometer permits dual threshold settings, such as for the Violet SSC and FITC channels [30].

The equipment was precleaned with 30 min of double-distilled water washing. The assays were performed with a maximum event rate of up to 2000 events/s at the lowest flow rate (10 µL/min) to avoid swarm effects. True MVs (TMVs) were defined as double-positive events in the annexin V vs. calcein dot plot and MV phenotyping by surface staining with monoclonal antibodies.

All intra-assay quality controls were used in accordance with regulations described elsewhere: annexin binding buffer-only control, buffer with reagents controls, unstained controls, single-stained controls, and fluorescence-minus-one (FMO) controls. The equivalent nanometric sizes of MVs were established by acquiring a mix of size-defined polystyrene beads, Megamix-Plus FSC (100, 300, 500, and 900 nm), and Megamix-Plus SSC (160, 200, 240, and 500 nm) (BioCytex, Marseille, France) through a nano-flow cytometric protocol comprising a Violet SSC-logA histogram, which showed 8-Gigamix-bead peaks with diameters ranging from 100 to 900 nm. All flow cytometry data analyses were performed using the CytExpert software version 2.4 (Beckman Coulter).

### 4.5. Scanning Electron Microscopy (SEM) Analysis

MVs were obtained as described above, and after the last centrifugation, the pellets were resuspended (50 μL) in 2.5% glutaraldehyde (Sigma-Aldrich, Burlington, MA, USA) in cacodylate buffer (0.1 M), pH 7.2, and the samples (10 μL) were adhered to glass coverslips previously covered with poly-L-lysine (Sigma-Aldrich). After 30 min at 37 °C, the coverslips were washed three times in cacodylate buffer and postfixed with a solution of 1% OsO_4_ containing 0.8% potassium ferrocyanide and 5 mM CaCl_2_ for 20 min at 25 °C. After being washed with the same buffer, the samples were dehydrated in an ascending series of ethanol (50, 70, 90, and 100%), dried using the critical point method, mounted on aluminum stubs, and finally coated with a 20 nm-thick gold layer and examined with a scanning electron microscope (Jeol JSM6390LV, Tokyo, Japan) at Rudolph Bar electron microscopy facility, IOC, FIOCRUZ. The size of the MVs was evaluated using the ImageJ program version 1.53m (U.S. National Institutes of Health, Bethesda, MD, USA).

### 4.6. Dynamic Light Scattering (DLS) and Electrophoretic Light Scattering (ELS)

Isolated plasma MVs were characterized by their size and stability (zeta potential) by DLS and ELS, respectively, with a Zetasizer Nano ZS90 (Malvern Panalytical, Malvern, UK). In brief, the isolated microvesicles were diluted in 1 mL PBS, and for DLS, the absorption of this suspension was measured at 632.8 nm three times in each sample. Zeta potential was also measured three times in each sample at 25°C with a frame rate of 10 frames per second. Zetasizer software version 7.13 (Malvern Panalytical) was used to collect and analyze the data.

### 4.7. Biochemical Analysis

The CK isoform from skeletal muscle (CK-NAC) was assessed in mouse plasma once per week during the three weeks of the swimming protocol. Blood samples were collected from tail snips in heparinized capillary tubes, centrifuged, and analyzed using commercially available kits (Wiener LaboratoriesRosario, AR) according to the manufacturer’s instructions. This quantitative assay was used as a marker of muscle damage.

Additionally, we evaluated CK-NAC in MV samples as an indicator of the origin of the MVs in skeletal muscle. For this assay, MV pellets were resuspended in lysis buffer (1% Nonidet P-40, 1 mmol/L leupeptin, 100 mmol/L PMSF, 1 mmol/L pepstatin A, and 100 mmol/L EDTA; all purchased from Sigma-Aldrich) before CK-NAC evaluation, which was performed as recommended by the manufacturer (Wiener Laboratories). The results are expressed as units per microgram of total protein per sample. Protein concentrations were determined using a bicinchoninic acid–based kit (Thermo Fisher) according to the manufacturer’s instructions.

### 4.8. Adoptive Transfer of Plasma MVs

Plasma MVs were obtained from 12-week-old *mdx* and DBA/2 mice as previously described. The MVs were quantified by flow cytometry as described here, and a total of 100 µL containing 1 × 10^8^ MVs was injected into the right tibialis anterior muscle paw of each animal. The following groups were prepared: *Mdx* mice that received MVs from *mdx* mice, *mdx* mice that received MVs from DBA/2 mice, DBA/2 mice that received MVs from DBA/2 mice, and DBA/2 mice that received MVs from *mdx* mice. The left tibialis anterior paw of each mouse received 100 µL of PBS, and a group of mice (*mdx* and DBA/2) that received no injection was also included (NI). Each group consisted of 4 animals. The animals were euthanized ten days after the injection, and the tibialis anterior muscles were collected and mounted on cork supports using gum tragacanth (Sigma-Aldrich). The samples were snap-frozen in isopentane cooled with liquid nitrogen and stored at --80 °C until use. Eight-micron-thick serial cross-sections were cut using a cryostat at --25 °C and stained with H&E. Slides were scanned using Motic Easy Scan with the software Motic DSAssistant version 3.0 (Motic, Hong Kong). For minimal Feret diameter evaluation, a total of 400 fibers per tissue section were measured, and the percentages of different diameter groups (<10 µm, 10–20 µm, 20–30 µm, 30–40 µm, 40–50 µm, 50–60 µm, 60–70 µm, >70 µm) were quantified. The percentage of centrally nucleated myofibers was determined in 1000 myofibers per animal in a total of 4 mice. The inflammatory area was determined by measuring the area of the total tissue slice divided by the area occupied by cellular inflammatory infiltrates in three slices per animal in a total of 4 mice. The minimal Feret diameter, percentage of the inflammatory area, and percentage of centrally nucleated myofibers were evaluated using the program ImageJ version 1.53m (U. S. National Institutes of Health, Stapleton, NY, USA).

### 4.9. Cytometric Bead Array (CBA)

Tibialis anterior fragments were incubated in ice-cold extraction buffer (1% Nonidet P-40, 1 mmol/L leupeptin, 100 mmol/L PMSF, 1 mmol/L pepstatin A, and 100 mmol/L EDTA; all purchased from Sigma-Aldrich) and centrifuged (500× *g*), after which the supernatants were frozen until use. TNF-α, IL-6, IL-10, IFN-γ, IL-12, and MCP-1 were detected using a Cytometric Bead Array Inflammation Kit (BD) according to the manufacturer’s instructions. Samples were acquired using a Cytoflex S flow cytometer (Beckman Coulter), and data analysis was performed using the Cytometric Bead Array analysis FCAP software version 3 (BD). All results are expressed as picograms per milligram of total protein per sample. Protein concentrations were determined using a bicinchoninic acid–based kit (Thermo Fisher) according to the manufacturer’s instructions.

### 4.10. Statistical Analysis

The distribution of all variables was assessed using the Shapiro–Wilk test. Comparisons between groups were performed using ANOVA for parametric distributions and the Kruskal–Wallis test for nonparametric distributions. All the statistical analyses were performed using GraphPad Prism version 8.1. Statistical significance is represented as * *p* ≤ 0.05.

## Data Availability

Research data are available in figures from the manuscript.

## Supplementary Materials

The following supporting information can be downloaded at: https://www.mdpi.com/article/10.3390/ijms26083499/s1.
