# Supplementary material for: Plasma Microvesicles May Contribute to Muscle Damage in the mdx Mouse Model of Duchenne Muscular Dystrophy
Source: Int J Mol Sci. 2025 Apr 8;26(8):3499. doi: 10.3390/ijms26083499 (PMC12026684; doi:10.3390/ijms26083499)
Supplement: Supplementary file 1 [file ijms-26-03499-s001.zip › ijms-2961446-supplementary.pdf]

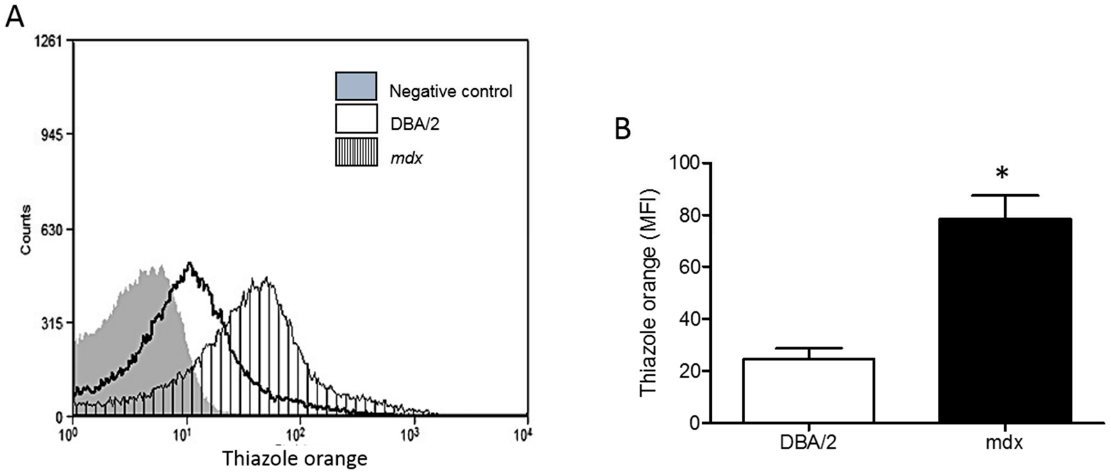


**Figure S1.** Nucleic acids evaluation in mdx mice plasma MVs; (A) Representative histogram of plasma MVs labeling of mdx and DBA/2 mice with thiazole orange As a negative control, plasma MVs from mdx mice not incubated with thiazole orange were used. (B) Median fluorescence intensity (MFI) of thiazole orange in plasma MVs of mdx and DBA/2 mice. * indicates $p < 0.05$ and The Kruskal–Wallis test, followed by Dunn's post hoc test, was used.
